# A novel pansharpening method based on cross stage partial network and transformer

**DOI:** 10.1038/s41598-024-63336-w

**Published:** 2024-06-02

**Authors:** Yingxia Chen, Huiqi Liu, Faming Fang

**Affiliations:** 1 School of Computer Science, Yangtze University, Jingzhou, 432023 China; 2 Shanghai Key Laboratory of Multidimensional Information Processing, East China Normal University, Shanghai, 200241 China; 3 School of Computer Science and Technology, East China Normal University, Shanghai, 200062 China

**Keywords:** Space physics, Engineering

## Abstract

In remote sensing image fusion, the conventional Convolutional Neural Networks (CNNs) extract local features of the image through layered convolution, which is limited by the receptive field and struggles to capture global features. Transformer utilizes self-attention to capture long-distance dependencies in images, which has a global receptive field, but the computational cost for high-resolution images is excessively high. In response to the above issues, this paper draws inspiration from the FusionNet network, harnessing the local detail acquisition capability of CNNs and the global data procuring capacity of Transformer. It presents a novel method for remote sensing image sharpening named Guided Filtering-Cross Stage Partial Network-Transformer, abbreviated as GF-CSTNet. This solution unifies the strengths of Guided Filtering (GF), Cross Stage Partial Network (CSPNet), and Transformer. Firstly, this method utilizes GF to enhance the acquired remote sensing image data. The CSPNet and Transformer structures are then combined to further enhance fusion performance by leveraging their respective advantages. Subsequently, a Rep-Conv2Former method is designed to streamline attention and extract diverse receptive field features through a multi-scale convolution modulator block. Simultaneously, a reparameterization module is constructed to integrate the multiple branches generated during training into a unified branch during inference, thereby optimizing the model’s inference speed. Finally, a residual learning module incorporating attention has been devised to augment the modeling and feature extraction capabilities of images. Experimental results obtained from the GaoFen-2 and WorldView-3 datasets demonstrate the effectiveness of the proposed GF-CSTNet approach. It effectively extracts detailed information from images while avoiding the problem of spectral distortion.

## Introduction

Pansharpening stands as a crucial technique in the processing of remote sensing images. Its objective is to merge low-resolution multispectral (MS) images with high-resolution panchromatic (PAN) images to generate multispectral images with a higher spatial resolution. The fused high space resolution multispectral (HRMS) images contain more information than the individual source pictures, thereby effectively compensating for the limited information in a single image. In applications such as object identification and image classification, pansharpening can provide high-quality images. Therefore, the pursuit of obtaining higher quality fused images has emerged as a prominent subject explored by numerous scholars.

Currently, there are two main types of representative sharpening approaches: deep learning algorithms and traditional algorithms. Traditional methods can be categorized into Component Substitution (CS), Multi-Resolution Analysis (MRA), and model-based approaches. Gram-Schmidt transformation (GS)^1^, Principal Component Analysis (PCA)^2^, and the Band-dependent Spatial Detail (BDSD)^3^, among other methods, are examples of common CS methods. These methods aim to substitute the space components of multispectral images with panchromatic images while retaining as much of the original spectral information as possible. While the aforementioned methods are user-friendly, the direct replacement approach may introduce a certain level of spectral distortion by destroying information in the image. The fundamental principle of Multi-Resolution Analysis (MRA) approaches entails subjecting the panchromatic (PAN) images to multi-scale transformations initially, and subsequently fusing them with the multispectral (MS) images. The commonly employed methods include the Discrete Wavelet Transform (DWT)^4^, Non-Subsampled Contour Transform (NSCT)^5^, Laplace Pyramid (LP)^6^, and various others. While these methods partially mitigate spectral distortion issues, they are prone to artifacts, and the resulting fusion may not reach an optimal outcome. Model-based methods treat pansharpening as a process of image reconstruction. They formulate and solve objective functions to reconstruct the fused image by utilizing features from previous iterations. Common methods encompass fusion techniques based on Variational Optimization (VO)^7^, Compressed Sensing-based methods^8^, and Bayesian-based fusion^9^, among others. Such methods exhibit fewer losses in spectral features and spatial details compared to CS and MRA methods. However, they require significant prior knowledge and involve complex algorithms, which prevent them from achieving the desired fusion results.

Because of their superior feature representation capabilities, deep learning techniques have become increasingly popular in domains such as remote sensing image fusion, driven by the rapid advancements in artificial intelligence in recent years. Inspired by super-resolution neural networks, Masi et al.^10^ introduced an initial CNN-based sharpening algorithm called PNN. This approach involves sending both the PAN images and the upsampled MS images to the network for processing. Subsequently, valuable information is extracted and combined from the images using a CNN. While this approach significantly improved performance, its simple 3-layer convolutional structure limited the model’s ability to capture nonlinearity, resulting in some spectral distortion. Subsequently, numerous advanced algorithms have been proposed. For instance, Yang et al.^11^ introduced PanNet, a sharpening approach based on deep residual networks. The utilization of residual learning enables the conversion of the network’s training process into the high-pass domain, which helps the network effectively learn high-frequency inputs. MSDCNN is a multi-scale, multi-depth CNN sharpening algorithm proposed by Yuan et al.^12^. It extracts features from diverse scale receptive fields by employing convolutional kernels of varying sizes. The combination of these features enhances the accuracy of feature map extraction. CNN was combined with conventional techniques to create the FusionNet network structure, as suggested by Deng et al.^13^. This method yields better results in both downscaling and full scaling by directly utilizing a singleness PAN image and every MS waveband to obtain image feature information. Wang et al.^14^ proposed a detail injection-based two-branch network, which utilizes the CS and MRA algorithms’ detail injection technique to directly learn the detail information of up-sampled MS images, thereby increasing the fusion image’s empty spectral quality. Fang et al.^15^ proposed SDRCNN, a lightweight CNN architecture with a single branch and scaling. This architecture is innovative because it incorporates a unique convolutional block and dense residual connection structure. This design preserves the spatial and spectral information of the image while striking a balance between accuracy and efficiency. A numerous scales multi-stream fusion network known as MMFN was proposed by Jian et al.^16^. The network first extracts a variety of features in MS and PAN images through a numerous scales strategy, and then fuses the extracted information with multi-stream fusion blocks to retain the optimal spatial and spectral characteristics. Que et al.^17^ effectively integrated spatial spectral information using a bilateral pyramid structure, achieving end-to-end fusion of multiple branches and inputs, and significantly improving the sharpening effect. Lu et al.^18^ proposed a novel self-guided spatial channel adaptive convolution technique called SSCAConv. This method adeptly considers a feature’s spatial and channel differences while maintaining an outstanding sharpening outcome. Furthermore, it can expand its application to address the super-resolution issue in hyperspectral remote sensing images, demonstrating its robust adaptability. Despite the significant advancements in CNN-based fusion techniques within the realm of remote sensing image fusion, challenges persist, such as limited receptive fields, insufficient capture of contextual information, and inadequate feature extraction.

This paper introduces the Transformer structure to address these issues, which can be viewed as a complement to the CNN model. This structure mitigates model biases and strengthens the ability to model remote dependencies^19,20^.

GF-CSTNet, a novel pansharpening approach that combines the Transformer and CSPNet networks, presents the following main contributions:

- The input data is preprocessed using the guided filtering method to preserve detailed information in the image.
- Combine the Transformer architecture with the CSPNet network, significantly enhancing the model’s learning capacity. This integration leverages the multi-branch features of CSPNet and the global modeling advantages of Transformer, leading to improved overall performance in the fused image.
- Design the Rep-Conv2Former module to simplify attention. Within this module, a reparameterization structure is constructed to not only reduce complexity but also extract more feature information from different receptive fields.
- Enhance the difference method. Introduce a residual learning module with attention to extract details from the image, creating a fusion image with a clear boundary.

## Related works

### FusionNet

FusionNet, a deep convolutional neural network that incorporates detail injection, was proposed by Deng et al.^13^. It was inspired by two classic sharpening methods: CS and MRA. The network adopts a differencing approach to gather meaningful information from the PAN and upsampled MS pictures. This message is then inputted into multiple residual blocks to further extract the image’s feature information. Finally, a fused image is produced by combining it with the upsampled MS images. The following is the precise implementation formula:

1 $$\begin{aligned} {\widehat{MS}} = {\widetilde{MS}} + f_{\Theta _{FS}} \left( P^D -{\widetilde{MS}} \right) . \end{aligned}$$

where ${\widehat{MS}}$ and ${\widetilde{MS}}$ are obtained by the superposition of ${\widehat{MS}}_i, i = 1, 2,..., B$ and ${\widetilde{MS}}_i, i = 1, 2,..., B$ bands, the high space resolution MS image of the *i*th band is represented by ${\widehat{MS}}_i$, ${\widetilde{MS}}_i$ denotes a lower space resolution MS image after upsampling the *i*th band, $P^D$ is the PAN image replicated across the spectral scale, and $f_{\Theta _{FS}}$ is the non-linear mapping function of FS.

The paper introduces a residual learning module that incorporates attention, based on the FusionNet model. It aims to extract edge information from images by employing a learn-then-subtract methodology, with detailed elaboration provided in section “Residual Learning”.

### CSPNet

Wang et al.^21^ presented a CNN model called CSPNet with the aim of enhancing the learning capacity and efficiency of the model. The basic idea of this network is to divide the feature mappings of the base layer into two halves. One part undergoes feature extraction through the nth ResNet blocks, while the other part combines the results extracted from the previous segment using cross-stage layers. Subsequently, tensor transformations and skip connections are used to generate significant differences in correlation within the backpropagation gradient flow. This design enables a significant reduction in the workload of processing feature maps while preserving some of the advanced characteristics of the feature graph, thereby improving the precision of the model. This design allows for a significant reduction in the workload of processing feature maps while preserving some of the advanced characteristics of the feature graph, thereby improving the precision of the model.

To strengthen the ability to be learned about the algorithm and enhance the fused images’ accuracy, the study integrates the CSPNet network structure into the technique of the remote satellite image.

### Attention mechanism

Presently, prevalent sequence transformation models mainly employ intricate RNN and CNN structures, comprising an encoder and a decoder. However, in constructing the model, it is crucial to consider not only the current output but also the output from the previous state. To achieve the ultimate goal, the network must perform calculations step by step. Additionally, transmitting data prematurely into the network may lead to incomplete content or even its discard. Even if this information is not discarded, it results in memory redundancy as the network continues to propagate. In response to this, Vaswani et al.^22^ introduced a novel Transformer architecture. This structure exclusively utilizes multi-head self-attention (MHSA) to compute implicit representations between the input and output of the model, significantly improving parallel processing capability and learning efficiency. However, this algorithm demands extensive computation when processing high-resolution images. To address this, Hou et al.^23^ proposed the Conv2Former method. This algorithm simplifies self-attention by employing the Hadamard product between the output and values of large-kernel convolutions, reducing the computational workload while enhancing efficiency.

The paper utilizes the Transformer structure to develop an algorithm for remote sensing image fusion. Meanwhile, drawing inspiration from the Conv2Former method and the RepVGG^24^ method. The Rep-Conv2Former attention block and the Rep-DConv reparameterization block were designed. These blocks aim to simplify multi-head self-attention and improve the efficiency of the model’s inference. Additionally, the incorporation of these additional structures enhances the model’s expressiveness and effectively overcomes the limitations of traditional convolutional neural networks in terms of receptive field size.

## Proposed method

**Figure 1:**
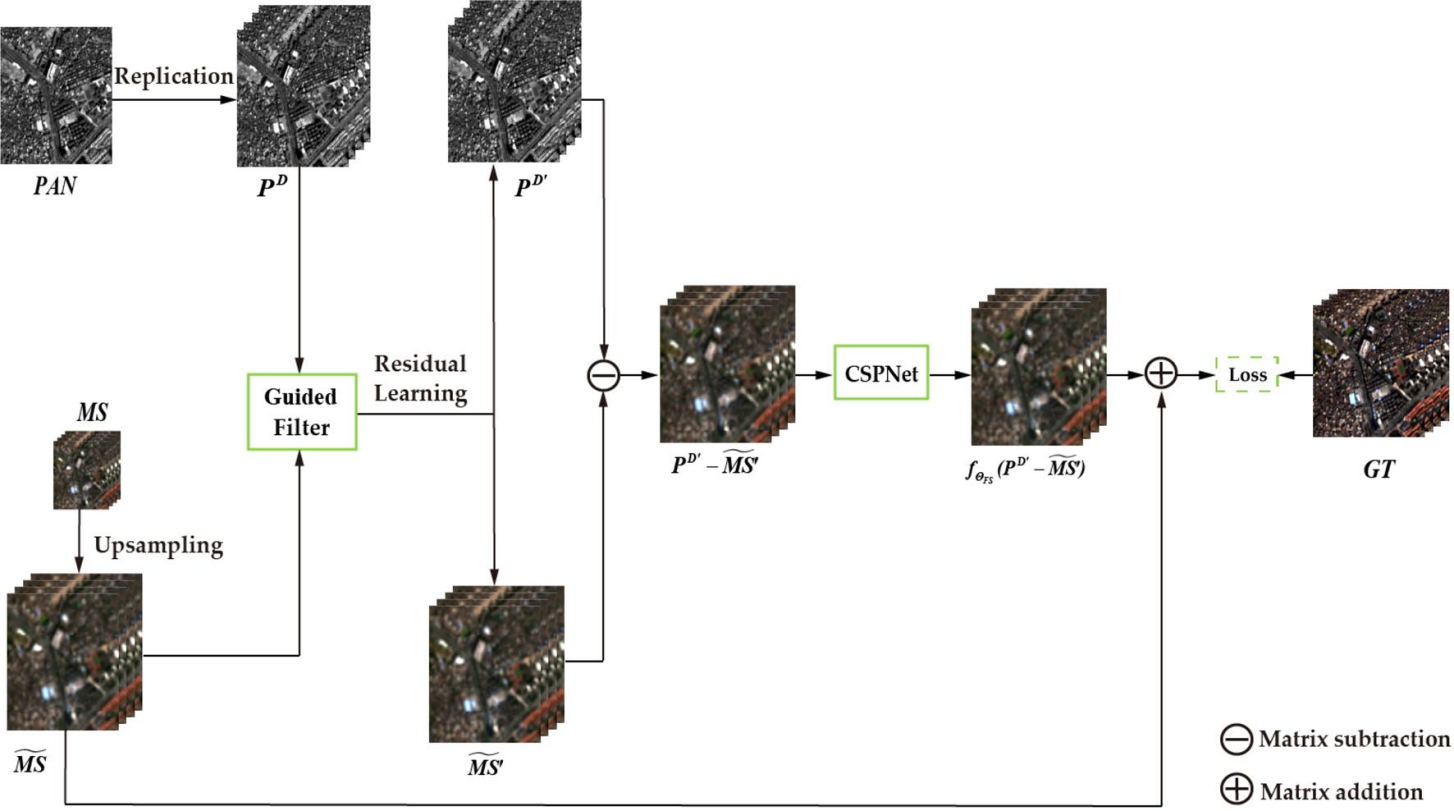
Integrated network overall structure diagram.

Figure 1 depicts the overall design of the fusion network, and each module will be explained in more detail in the following sections.

### Guided filtering

Guided filtering^25^ is a technique for image filtering that is based on a local linear model. The fundamental idea is to locally linearly filter a guidance image, implying the filtering of an input image using the guidance image. In this way, the texture information of the guided image can be obtained while preserving the attributes of the input image. By employing this technique, it becomes possible to effectively remove noise from remote sensing image data and enhance the detailed information in the image. The detailed design procedure is outlined below: the upsampled MS image is labeled as the filtered input image L, the PAN image is labeled as the guide image P, and the filtered output image is labeled as H. The principle of guided filtering can be expressed as follows:

2 $$\begin{aligned} H_i = a_kP_i + b_k, \forall i \in \omega _k, \end{aligned}$$

where *i* and *k* represent pixel indices, $\omega _k$ is the *k*th local window, the numerical value for the *i*th pixel in the filtered output image H is represented by $H_i$, $P_i$ is a representation the magnitude of the *i*th pixel in the guide image P, $a_k$ and $b_k$ are the linear coefficients calculated by the filtered output image $H_i$ within window $\omega _k$. This can be achieved by minimizing the distinction between the input image P and the output graph H. The formula is expressed as follows:

3 $$\begin{aligned} E = (a_k, b_k) = \sum _{\begin{array}{c} i \in \omega _k \end{array}} \left( \left( a_kP_i + b_k - L_i \right) ^2 + \varepsilon a_k^2 \right) . \end{aligned}$$

In the filtered input image L, where $L_i$ represents the measurement of the *i*th pixel, $\varepsilon$ is a regularization parameter aimed at preventing excessively large $a_k$ coefficients that could affect the filtering effect. In equation (3), the optimum amounts for the coefficients of linearity $a_k$ and $b_k$ are estimated by optimizing the fitting value $E(a_k, b_k)$ . The function is expressed as follows and can be obtained through the least squares approach.

4 $$\begin{aligned} a_k= & {} \frac{\frac{1}{\left| \omega \right| } \sum _{\begin{array}{c} i \in \omega _k \end{array}} P_iL_i-\mu _k{\overline{L}}_k}{\sigma _k^2+\varepsilon }, \end{aligned}$$

5 $$\begin{aligned} b_{k}= & {} {\overline{L}}_k-a_{k} \mu _{k}. \end{aligned}$$

where $\left| \omega \right|$ denotes all the pixels in the window $\omega _k$, the guide picture inside the window is represented by $\mu _{k}$ and $\sigma _k^2$, respectively, as its average and variance. ${\overline{L}}_k$ represents the average pixel value for the filtered input image L within the window. However, during the calculation of linear coefficients for each window, it is discovered that pixel *i* could be included in multiple windows $\omega _k$ . In other words, each pixel is described by multiple distinct linear functions. This implies that the filtered output image $H_i$ in Eq. (2) continuously varies with the alteration of $\omega _k$ . For this purpose, the mean method is adopted to calculate the average of the values within the window of each pixel point *i*. The ultimate result graph of the filter can be represented as follows:

6 $$\begin{aligned} H_i=\frac{1}{\left| \omega \right| }\sum _{i\in \omega _k}(a_kP_i+b_k)={\overline{a}}_iP_i+{\overline{b}}_i. \end{aligned}$$

where $\overline{a_i}=\frac{1}{\left| \omega \right| }\sum _{k\in \omega _i}a_k, \overline{b_i}=\frac{1}{\left| \omega \right| }\sum _{k\in \omega _i}b_k$ represent the mean coefficients, which are the average values of the coefficients across all windows that include pixel *i*.

From the above process, it can be observed that the GF algorithm demonstrates a strong local correlation. By utilizing the window average and linear invariance transformation model, it is possible to incorporate specific details from the reference graph P into the input image L while preserving the overall characteristics of the input graph L, in order to obtain the output image H with enhanced visual impact. In this manner, the minimization of the difference between P and L is pursued to the greatest extent, leading to a higher-quality image for subsequent fusion.

### Backbone network enhancement

Feature graphs at different levels can be concatenated using CSPNet to optimize network performance when integrated with various convolutional neural network architectures. Therefore, in this section, the backbone network is constructed by combining CSPNet and ResNet. Additionally, this network includes an Attention ResBlock based on Convolutional Transformer. This design leverages the high-performance characteristics of multi-branch networks and the advantages of global modeling in Transformers, further augmenting the fusion capabilities of the model.

**Figure 2:**
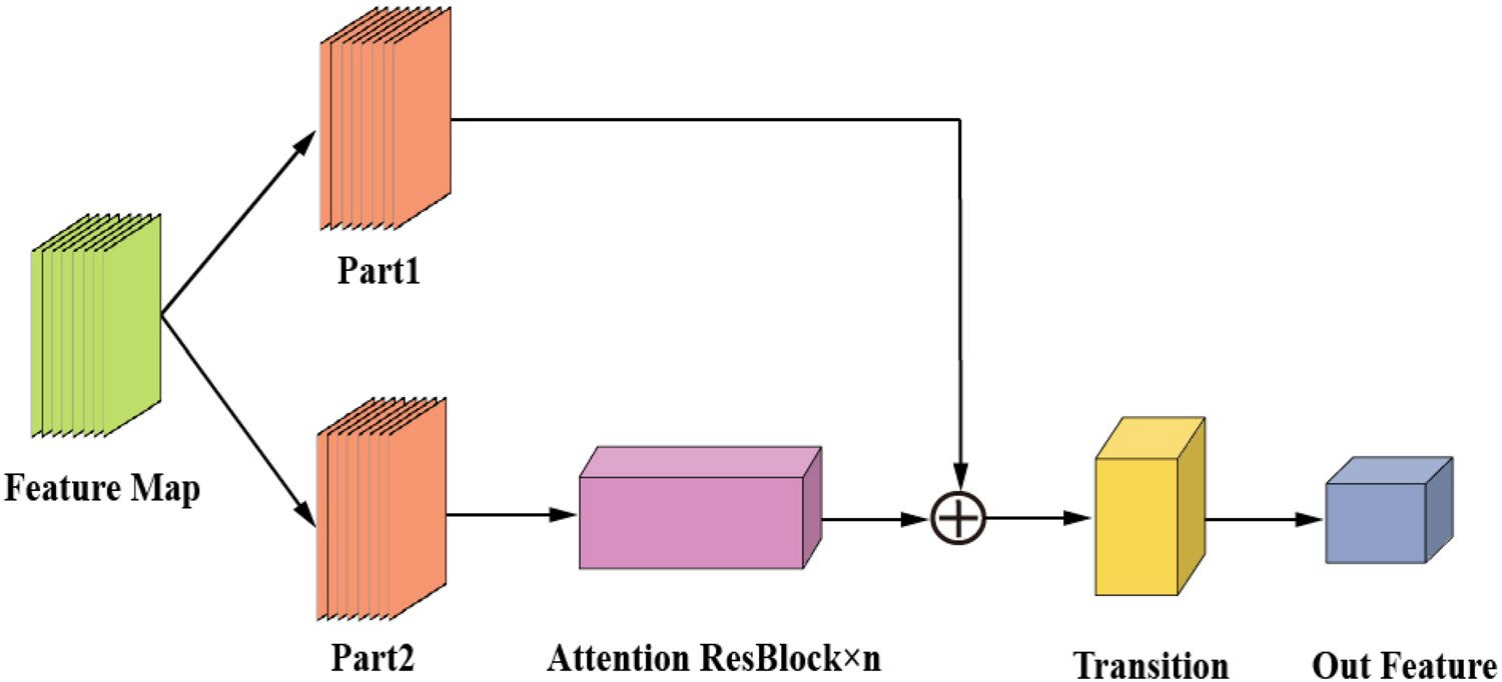
Backbone network structure diagram.

**Figure 3:**
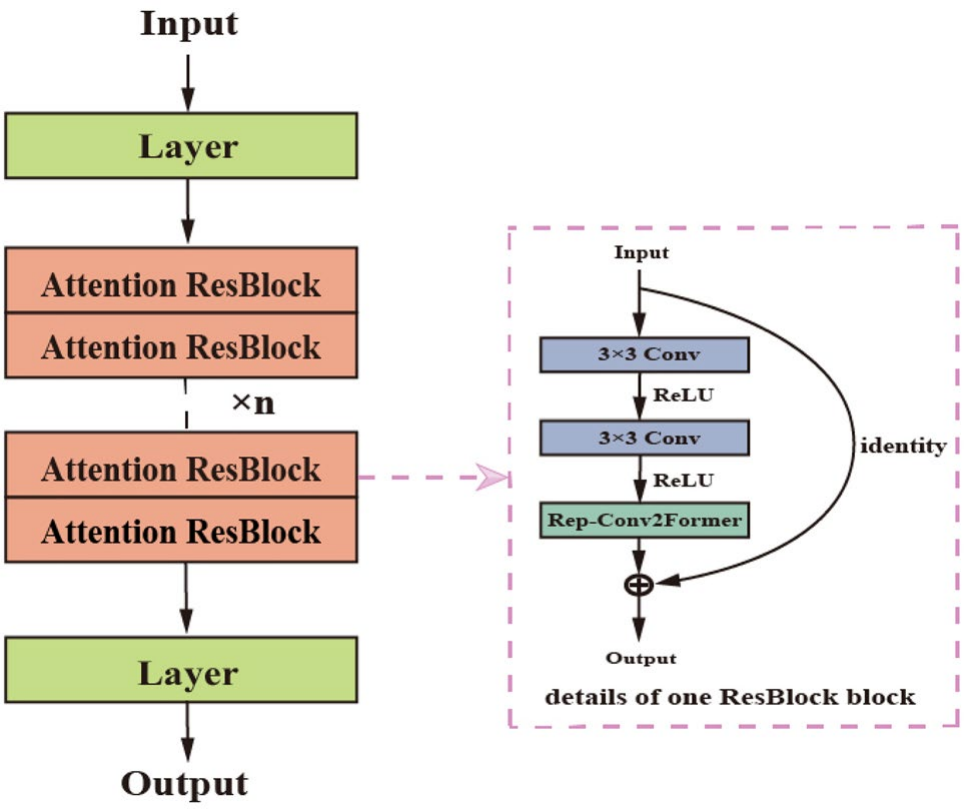
Structure diagram of multiple attention ResBlock blocks.

Figure 2 shows the backbone network’s general architecture, with a more detailed depiction of the Attention ResBlock in Fig. 3. The main idea is as follows: Firstly, the feature graph $P^{D^{\prime }} - \widetilde{MS^{\prime }}$, extracted by the residual learning module, is set as $(P^{D^{\prime }} - \widetilde{MS^{\prime }})_0$ . Then, it is divided into two branches, Part 1: $(P^{D^{\prime }} - \widetilde{MS^{\prime }})_0^{\prime }$ and Part 2: $(P^{D^{\prime }}-\widetilde{MS^{\prime }})_0^{\prime \prime }$ . Part 2 performs deep-level feature extraction using *n*th Attention ResBlock blocks and then undergoes gradient boosting through the Transition layer. Finally, it is combined with Part 1 to achieve cross-stage splitting and merging. Employing this cross-stage structure not only enhances fusion performance but also enables the model to demonstrate greater adaptability and flexibility in complex network environments.

Based on this, the output of ResNet integrated into CSPNet is represented as follows:

7 $$\begin{aligned} \left( P^{D^{\prime }}-\widetilde{MS^{\prime }} \right) _K= & {} \omega _K* \left[ \left( P^{D^{\prime }}-\widetilde{MS^{\prime }} \right) _0^{\prime \prime }, \left( P^{D^{\prime }}-\widetilde{MS^{\prime }} \right) _1, \left( P^{D^{\prime }}-\widetilde{MS^{\prime }} \right) _2,\cdots \left( P^{D^{\prime }}-\widetilde{MS^{\prime }} \right) _{K-1} \right] , \end{aligned}$$

8 $$\begin{aligned} \left( P^{D^{\prime }}-\widetilde{MS^{\prime }} \right) _T= & {} \omega _T* \left[ \left( P^{D^{\prime }}-\widetilde{MS^{\prime }} \right) _0^{\prime \prime }, \left( P^{D^{\prime }}-\widetilde{MS^{\prime }} \right) _1, \left( P^{D^{\prime }}-\widetilde{MS^{\prime }} \right) _2,\cdots , \left( P^{D^{\prime }}-\widetilde{MS^{\prime }} \right) _K \right] , \end{aligned}$$

9 $$\begin{aligned} \left( P^{D^{\prime }}-\widetilde{MS^{\prime }} \right) _Z= & {} \omega _Z* \left[ \left( P^{D^{\prime }}-\widetilde{MS^{\prime }} \right) _0^{\prime }, \left( P^{D^{\prime }}-\widetilde{MS^{\prime }} \right) _T \right] . \end{aligned}$$

where $*$ represents convolution, $\omega$ represents the weight, and $(P^{D^{\prime }}-\widetilde{MS^{\prime }})_K$ denotes the output of the *K*th Attention ResBlock, which is then converted into $(P^{D^{\prime }}-\widetilde{MS^{\prime }})_T$ and $(P^{D^{\prime }}-\widetilde{MS^{\prime }})_Z$ . The equation for the inverse update weight can be represented as follows:

10 $$\begin{aligned} \omega _K^{\prime }= & {} f \left( \omega _K,g_0^{\prime \prime },g_1,g_2,\cdots ,g_{K-1} \right) , \end{aligned}$$

11 $$\begin{aligned} \omega _T^{\prime }= & {} f \left( \omega _T,g_0^{\prime \prime },g_1,g_2,\cdots ,g_K \right) , \end{aligned}$$

12 $$\begin{aligned} \omega _Z^{\prime }= & {} f \left( \omega _Z,g_0^{\prime },g_T \right) . \end{aligned}$$

where $g_K$ represents the gradient of the *K*th Attention ResBlock, and *f* denotes the weight update function.

In conclusion, CSPNet is a simple and versatile network, combining ResNet with the Convolutional Morphology Transformer and designing the Attention ResBlock blocks as the backbone network. By implementing this approach, the problem of reusing gradient information is effectively avoided, resulting in improved overall performance of the model. Consequently, it successfully attains the goal of improving the spectral and spatial resolution of the fused image.

### Attention simplification

As demonstrated in the previous text, incorporating a Transformer block into the network can improve the model’s learning capacity. However, the application of multi-head self-attention increases computational complexity. In this section, a Rep-Conv2Former module is designed to address this issue. The approach draws inspiration from the Conv2Former method and strives to optimize attention. In contrast to the reference method, this module extracts features from different receptive fields using multi-scale convolutions (Conv 3 $\times$ 3, Conv 11 $\times$ 11) to integrate and leverage global information at various scales. Simultaneously, the Rep-Conv2Former module integrates a Rep-DConv reparameterization block, drawing inspiration from the multi-branch merging concept of RepVGG and its associated transformation method. Employing the concept of structural reparameterization, the multi-way (Conv 3 $\times$ 3, Conv 11 $\times$ 11) structure of the training network is transformed into a single-way (Conv 11 $\times$ 11) structure for the inference network. This transformation significantly enhances the network’s inference rate.

**Figure 4:**
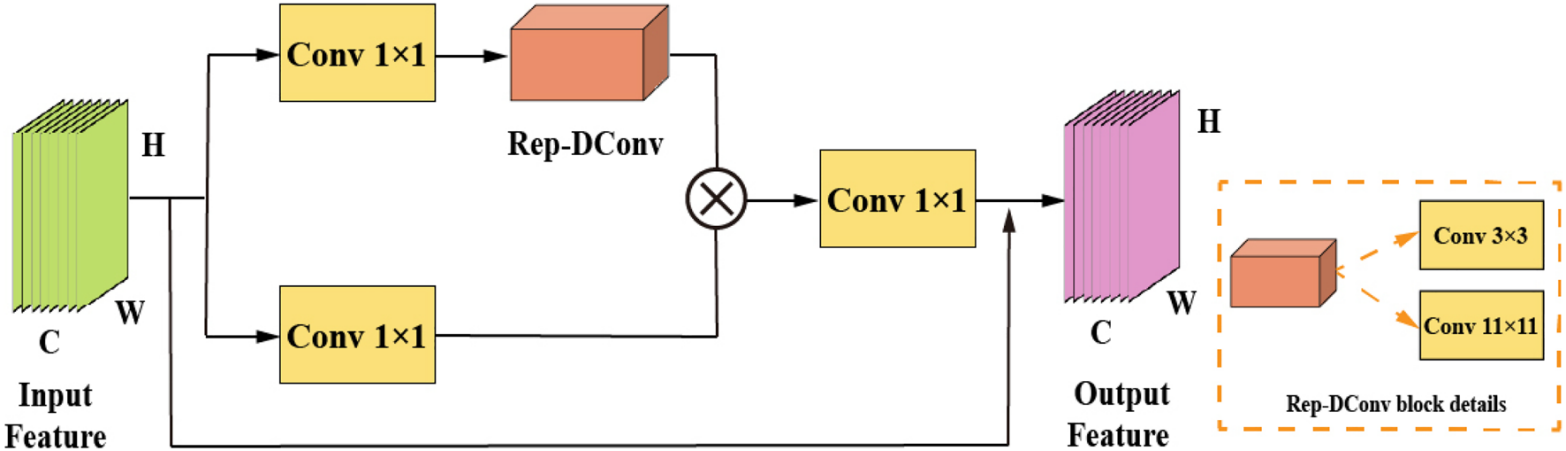
Structure diagram of the Rep-Conv2Former method.

The structure is illustrated in Fig. 4, and detailed explanations will be provided below.

#### Rep-Conv2Former

**Figure 5:**
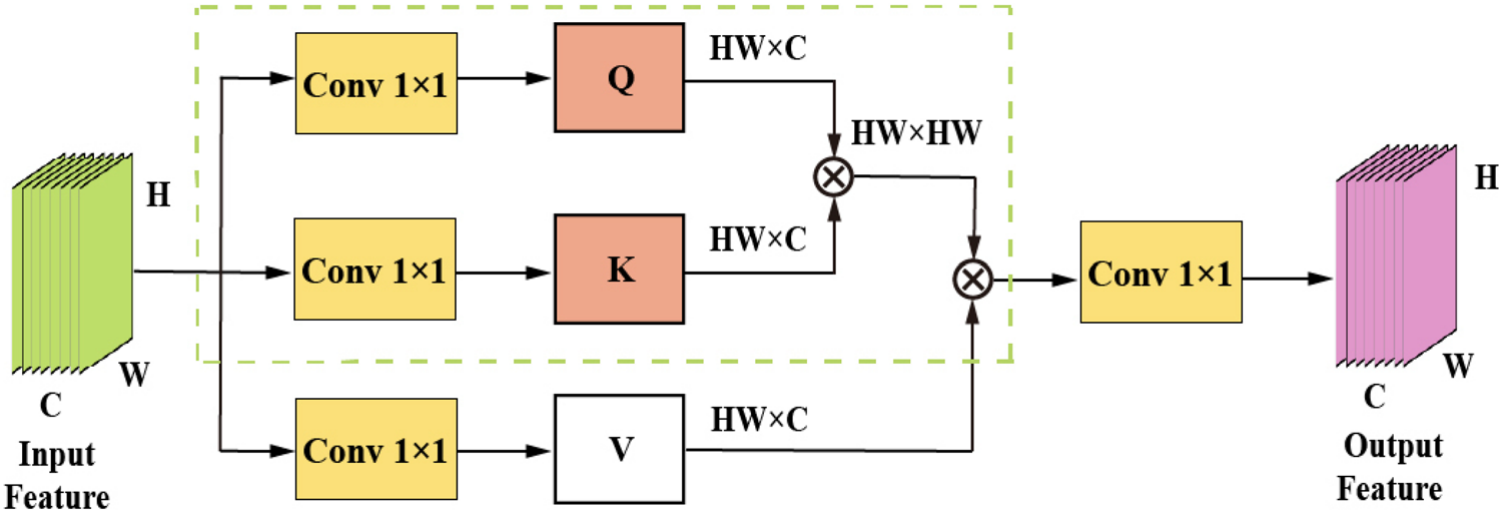
Self-attention structure diagram.

This subsection will outline the convolutional modulation block used in Rep-Conv2Former. Firstly, the self-attention block is compared to the convolutional modulation block. From Fig. 5, assuming the Part 2 branch feature graph $(P^{D^{\prime }}-\widetilde{MS^{\prime }})_0^{\prime \prime }$ with a size of H $\times$ W $\times$ C, extracted by subtraction, is taken as the input. The self-attention first undergoes a correlation operation through a linear layer to obtain the query matrix **Q**, key matrix **K**, and value matrix **V**. Among them, ${\textbf{K}},{\textbf{Q}},{\textbf{V}}\in {\mathbb {R}}^{H\times W\times C}$, *C* represents the overall number of channels, while *H* and *W* represent the input’s spatial dimensions. The output is represented by the attention matrix **A**. The utilization of multi-head attention allows for learning information from different spatial locations, as demonstrated below.

13 $$\begin{aligned} \textrm{Attention} \left( \left( P^{D^{\prime }}-\widetilde{MS^{\prime }} \right) _0^{\prime \prime } \right) =\textbf{AV}, \end{aligned}$$

It is further rewritten as:

14 $$\begin{aligned} {\textbf{A}}=\textrm{Softmax} \left( \textbf{QK}^\top \right) . \end{aligned}$$

In the above equation, the scale factor is omitted for simplicity. Although the encoding of spatial information is highly efficient, its computational complexity increases as the size of the input feature graph grows, which leads to higher computational requirements.

**Figure 6:**
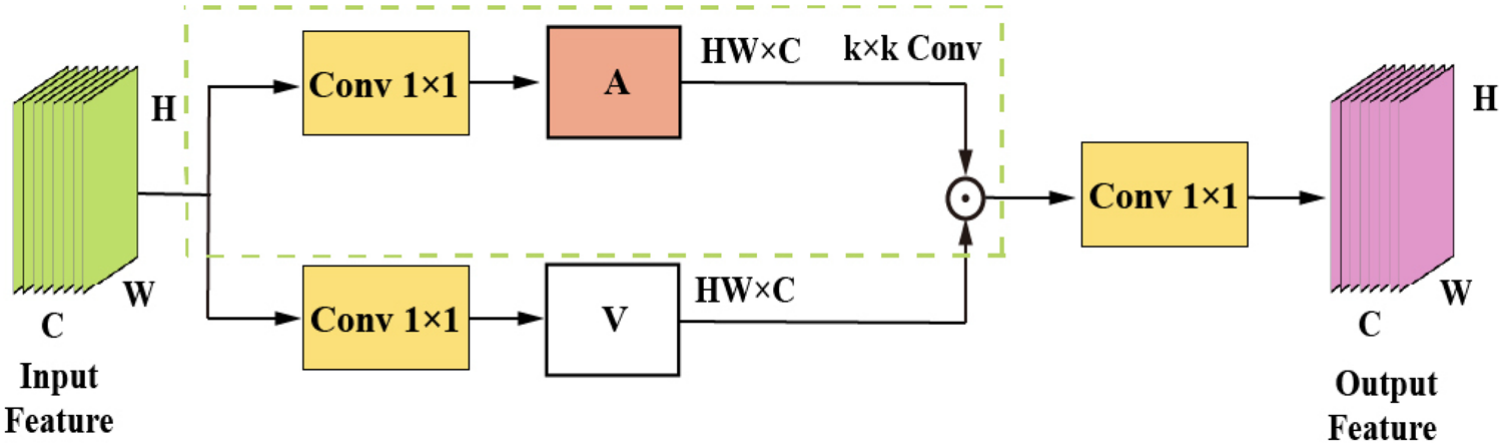
Convolutional modulation block structure diagram.

In Fig. 6, the convolution modulation block’s input $(P^{D^{\prime }}-\widetilde{MS^{\prime }})_0^{\prime \prime }\in {\mathbb {R}}^{H\times W\times C}$ is the Part 2 branch feature map extracted by the residual learning module. Convolution modulation, on the other hand, utilizes multi-scale convolution ${\text {Re}}\text {p-DConv}_{k\times k}$ of size *k* $\times$ *k* and the Hadamard product to determine the value of the output feature map **Z**, as depicted below.

15 $$\begin{aligned} {\textbf{Z}}= & {} {\textbf{A}}\odot {\textbf{V}}, \end{aligned}$$

16 $$\begin{aligned} {\textbf{A}}= & {} {\text {Re}}\text {p-DConv}_{k\times k} \left( {\textbf{W}}_1 \left( P^{D^{\prime }}-\widetilde{MS^{\prime }} \right) _0^{\prime \prime } \right) , \end{aligned}$$

17 $$\begin{aligned} {\textbf{V}}= & {} {\textbf{W}}_2 \left( P^{D^{\prime }}-\widetilde{MS^{\prime }} \right) _0^{\prime \prime }. \end{aligned}$$

where ${\text {Re}}\text {p-DConv}_{k\times k}$ can be expressed in detail as follows:

18 $$\begin{aligned} {\text {Re}}\text {p-DConv}_{k\times k}={\text {DConv}}_{k_1k_1}+{\text {DConv}}_{k_2k_2}. \end{aligned}$$

In the equation, $\odot$ represents the Hadamard product, the two linear layers’ parameters are denoted by $W_1$ and $W_2$, and *k* represents the reparameterized convolutional kernel. Here, $k_1k_1$ and $k_2k_2$ represent Conv 3 $\times$ 3 and Conv 11 $\times$ 11, respectively. By modulating the **V** values with this multi-scale convolution, not only can the characteristics of receptive fields of different sizes be generated, but also the position of space (*h*, *w*) can be linked to pixels in the *k* $\times$ *k* square area centered on (*h*, *w*) . Furthermore, channel-wise information exchange can be performed using linear layers.

In summary, self-attention employs matrix multiplication between **K** and **Q** to generate the attention matrix. Convolutional modulation utilizes multi-scale convolutions of size *k* $\times$ *k* to generate weights for the input’s feature map. The Hadamard product is then used to establish dependency relationships among features. By doing so, the model not only enhances the weights of high-frequency spatial features and spectral distribution features, but also extracts more profound feature information, thereby further improving its effectiveness.

#### Rep-DConv

RepVGG is a simple and efficient CNN architecture that mainly consists of an identity branch, 1 $\times$ 1 convolution, and 3 $\times$ 3 convolution. This method employs different structures during training and inference. Specifically, it utilizes a 3 $\times$ 3 convolution during training and incorporates a 1 $\times$ 1 convolution branch, as well as a branch for identity mapping. In reasoning, the concept of re-parameterization is employed to convert the model into a 3 $\times$ 3 convolutional branch. In contrast to this method, this section only utilizes the concept of multi-branch merging and the corresponding transformation method. Additionally, a Rep-DConv block is designed to improve computational speed.

**Figure 7:**
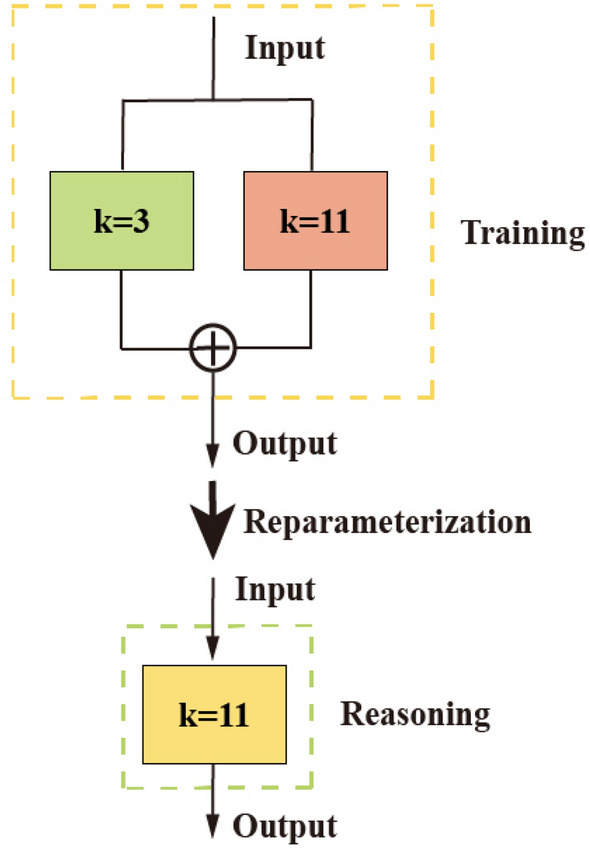
Training and reasoning diagram.

The structure of training and reasoning is shown in Fig. 7. After convolutional modulation, the feature maps in the model prioritize precision during the training phase. At this stage, the main components are a 3 $\times$ 3 convolution and an 11 $\times$ 11 convolution. Parallelizing the two branches to introduce multiple gradient paths to the network enhances the extraction and fusion of information at different scales in the feature map. This, in turn, further improves the model’s representational capacity. During the inference phase, the model prioritizes speed. The two training branches are combined into a single 11 $\times$ 11 convolutional branch through reparameterization. This enables the network to efficiently infer information while also acquiring the weights of the multi-branch training parameters.

In essence, this module employs structural reparameterization to convert the trained multi-branch structure into a single-branch architecture during inference. By merging the benefits of multi-branch high performance with single-branch fast speed, the model retains robust feature extraction capabilities while achieving rapid recognition speed.

### Residual learning

The FusionNet model extracts image characteristics through $P^D-{\widetilde{MS}}$, as can be observed from Eq. (1). However, there are instances where employing a direct subtraction method might not effectively emphasize the boundary information in the image. Therefore, in this section, a residual learning module incorporating attention is designed to address this issue.

The dashed area in Fig. 3 represents the specific architecture. The implementation process can be described as follows: the filtered image is obtained through the learning process of a residual block with attention. Every residual block is composed of two branches: the residual branch, which learns the distinction between the input data and the output, and the identity branch, which directly transfers the input to the output using an identity mapping. By incorporating these two branches, the residual learning results in $P^{D^{\prime }}$ and $\widetilde{MS^{\prime }}$ can be obtained. The obtained results are then subtracted to improve the image details’ clarity. The specific formula is represented as follows:

19 $$\begin{aligned} {\widehat{MS}}={\widetilde{MS}}+f_{\Theta _{FS}} \left( P^{D^{\prime }}-\widetilde{MS^{\prime }} \right) . \end{aligned}$$

where $P^{D^{\prime }}=f_1(P^D)$, $\widetilde{MS^{\prime }}=f_2({\widetilde{MS}})$, $f_1$ and $f_2$ represent the mapping layers of the two residuals.

Overall, the attention method is introduced in the residual learning module, where the process involves performing residual learning first and then subtracting the learned results. By leveraging the dual advantages of convolutional and Transformer models, the network becomes better at preserving the original input information while also learning complex features. This enhances the network’s ability to extract and represent image features, addressing the issue of vanishing gradients.

### Improvement of the loss function

During the training of the model, the loss function, which compares predicted values with actual values to evaluate the model’s performance, is calculated for each sample. Initially, the model utilizes forward propagation to generate a prediction. The difference between the anticipated and real outcomes is then calculated using the loss function. After obtaining the deviation, the model parameters are adjusted through backpropagation. This process aims to align the model’s predicted outcomes with the actual circumstances, thereby improving the validity of the predictions.

The FusionNet model utilizes the L2 loss function for network optimization. However, during practical training, it has been observed that overfitting occurs, where the model’s error during training is significantly lower than the error during testing. While increasing the amount of training samples may help reduce overfitting, it is more expensive to obtain additional training samples. Therefore, weight decay is used in this part to alleviate the overfitting problem. Weight decay penalizes models with large absolute values by adding a penalty term, which imposes constraints on the models that need to be learned. In this way, the model’s capacity for generalization is further improved while reducing overfitting. The formula is as follows:

20 $$\begin{aligned} \textrm{Loss}(\Theta _{_{FS}})=\frac{1}{n}\sum _{k=1}^{n}\left\| {\widetilde{MS}}_{\{k\}}+f_{\Theta _{_{FS}}}\left( P_{\{k\}}^{D}{}^{\prime }-{\widetilde{MS}}^{\prime }_{\{k\}}\right) -GT_{\{k\}}\right\| _{F}^{2}+\frac{\lambda }{2}{\left\| \Theta _{_{FS}}\right\| ^{2}} \end{aligned}$$

where *n* is the amount of training samples, the number *k*th from GT is denoted by $GT_{\{k\}}$, $\left\| \cdot \right\| _F$ is the Frobenius norm, $\lambda$ is a hyperparameter used to control the strength of weight decay.

### Fusion algorithm

Based on the aforementioned research content, the specific fusion algorithm can be expressed as follows:

- The LMS picture can be obtained by first duplicating the PAN image channel and then resizing the MS image to match the dimensions of the PAN image.
- The guided filtering method is applied using the PAN picture as the guide image, and the LMS image is used as the input map for image preprocessing.
- The filtered image is subtracted by the residual learning module to extract detailed information.
- The obtained information is fed into a two-branch CSPNet, which consists of Attention ResBlock blocks based on the convolutional form of Transformers. In this structure, Part 1 consists of only one layer of convolution, while Part 2 leverages the global modeling capability of the Transformer to extract more detailed information from the image. Next, it calculates the nth Attention ResBlock blocks to segment different gradient flows.
- Then, part 1 and part 2 are connected, and the result is inputted into a new convolutional layer to achieve the fusion of feature information.
- The fusion result is then output by connecting it to the input feature map through a skip connection.
- Finally, the output result undergoes non-linear mapping, and a skip connection is utilized to merge it with the LMS in order to generate the ultimate fusion image.

## Experimental validation and analysis

### Experimental preparation and dataset

#### Comparison methods and evaluation metric

This article selected several representative methods for comparative experiments, including EXP^26^ (multispectral images upsampled by 23-tap polynomial interpolation), BT-H^27^, BDSD-PC^28^, SR_D^29^, TV^30^, PNN^10^, BDPN^31^, MSDCNN^12^, DRPNN^32^, DiCNN1^33^, and FusionNet^13^. The comparison experiments in the paper were conducted in the same equipment and environment. The deep learning method adopted Pytorch framework and trained on GeForce RTX 4060, and the test used MATLAB (2019 a). Using the Adam optimizer during the training, the proposed method sets the initial rate of learning to 3e-4, weight decay to 1e-7, and the number of batches to 32. At 400 iterations, stable performance was achieved.

Both subjective and objective analyses of the fusion results are performed in the paper. Subjectively, fusion images are analyzed through direct observation of fusion results and local amplification. Objectively, eight commonly used evaluation metrics are employed to systematically analyze the fused images. These eight indicators are SAM^34^, ERGAS^34^, Q^34^, RMSE^34^, SID^35^, Q4/Q8^36,37^, RASE^34^, and SCC^34^.

#### Dataset preprocessing

The WorldView-3 (WV3) and GaoFen-2 (GF2) datasets were used in the experiment to demonstrating how effective to demonstrate the effectiveness of the strategy suggested in this study. However, the research will preprocess the GF2 and WV3 datasets using the GF technique to improve the fusion accuracy of the suggested approach. (Dataset download address: PanCollection for Survey Paper (liangjiandeng. github. io)).

### Fusion results analysis

#### GF2 dataset

**Figure 8:**
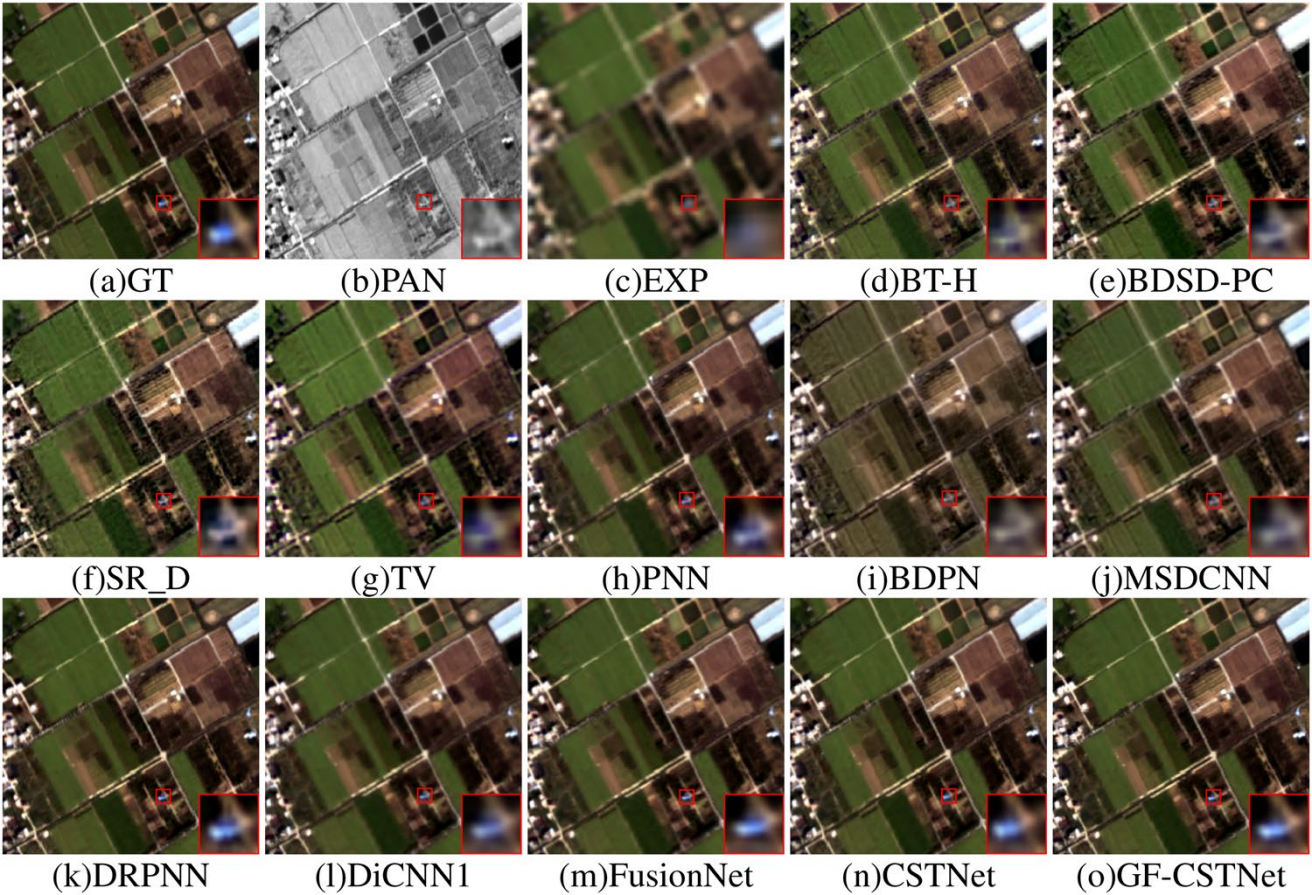
Sharpening results of test Fig. 1 on GF2 dataset.

**Figure 9:**
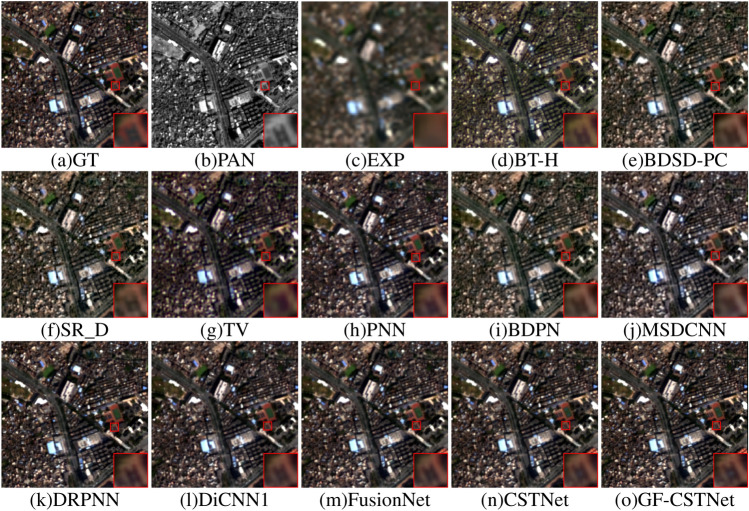
Sharpening results of test Fig. 2 on GF2 dataset.

Figures 8, 9 demonstrates the fusion renderings of both sets of remote sensing images that were tested on the GF2 dataset using different algorithms. Analyzed from a subjective standpoint, the fusion results of conventional algorithms such as BT-H (Fig. d), BDSD-PC (Fig. e), SR_D (Fig. f), and TV (Fig. g) exhibit pseudo-shadow phenomena in certain areas when compared to the Ground Truth (GT). The fusion outcomes of the PNN algorithm (Fig. h) show a significant improvement when it comes to space and spectrum properties compared to those of the traditional method. However, they still do not match the sharpness and minutiae of GT images in terms of visual quality. The result produced by the BDPN algorithm (Fig. i) exhibits a significant issue of spectral distortion, which leads to a loss of detail and an overall darkening of the image’s color. The MSDCNN algorithm (Fig. j) suffers from spectral distortion and performs poorly in terms of spatial quality, resulting in a relatively blurry fusion outcome. The DRPNN method (Fig. k), DiCNN1 algorithm (Fig. l), and FusionNet algorithm (Fig. m) all produce fusion results that successfully retain the image’s spectrum characteristics. Furthermore, the quality of space has also improved significantly. However, from the perspective of local amplification, there is some loss of information. CSTNet (Fig. n) and GF-CSTNet (Fig. o) are slightly superior to other comparison methods in regard to both space and spectrum detail. However, compared to CSTNet (without guided filter processing), the suggested method GF-CSTNet contains more spectrum information and distinct texture details. It achieves a higher spatial resolution and is more consistent with the GT image.

Tables 1, 2 present the objective evaluation metrics obtained by each algorithm across the GF2 dataset. The optimal values are indicated in bold. The conventional BT-H, BDSD-PC, SR_D, and TV algorithms exhibit relatively high values for ERGAS, RMSE, SID, and RASE metrics, as clearly indicated in the table, while showing the smallest values for SCC. This suggests that the information included in the original photos is not effectively captured by the fused images generated by these algorithms. They also exhibit lower spatial quality and spectral characteristics. The PNN, DRPNN, DiCNN1, and FusionNet algorithms have achieved favorable results across various metrics, indicating that the fusion results have been significantly improved in the corresponding spectrum and space realms. While the metrics of the CSTNet method outperform those of the other comparison approaches, they still fall short of the performance achieved by the GF-CSTNet method. This suggests that the outcomes of fusion can still be improved. The proposed GF-CSTNet method outperforms all other algorithms in every evaluation indicator. The fusion results exhibit a higher level of spatial detail and spectral fidelity, making the overall effect ideal.

**Table 1:** Evaluation indicators for sharpening results in Test Fig. 1.

Method	Evaluation Indicators							
	Q $\uparrow$	ERGAS $\downarrow$	Q4 $\uparrow$	RMSE $\downarrow$	SID $\downarrow$	SAM $\downarrow$	RASE $\downarrow$	SCC $\uparrow$
BT-H	0.8689	1.3813	0.8488	15.4721	0.0036	1.4779	5.1273	0.9067
BDSD-PC	0.8782	1.4361	0.8479	15.9795	0.0037	1.5082	5.2954	0.9095
SR_D	0.8972	1.2866	0.8703	14.2611	0.0058	1.3122	4.7260	0.9168
TV	0.8684	1.4433	0.8361	16.0584	0.0045	1.4892	5.3216	0.9172
PNN	0.9307	1.0042	0.9167	11.1021	0.0017	1.0165	3.6791	0.9578
BDPN	0.8665	1.4496	0.8316	15.9639	0.0044	1.9150	5.2903	0.9346
MSDCNN	0.8929	1.2223	0.8714	13.5643	0.0030	1.3528	4.4951	0.9401
DRPNN	0.9607	0.7345	0.9477	8.0284	0.0012	0.8495	2.6605	0.9741
DiCNN1	0.9477	0.8543	0.9318	9.3982	0.0015	0.9399	3.1145	0.9682
FusionNet	0.9554	0.8122	0.9399	8.9255	0.0013	0.8455	2.9578	0.9717
CSTNet	0.9675	0.6996	0.9583	7.5242	0.0009	0.7591	2.4935	0.9774
GF-CSTNet	**0.9701**	**0.6468**	**0.9586**	**7.0334**	**0.0008**	**0.7393**	**2.3308**	**0.9805**

Significant values are in bold.

#### WV3 dataset

**Figure 10:**
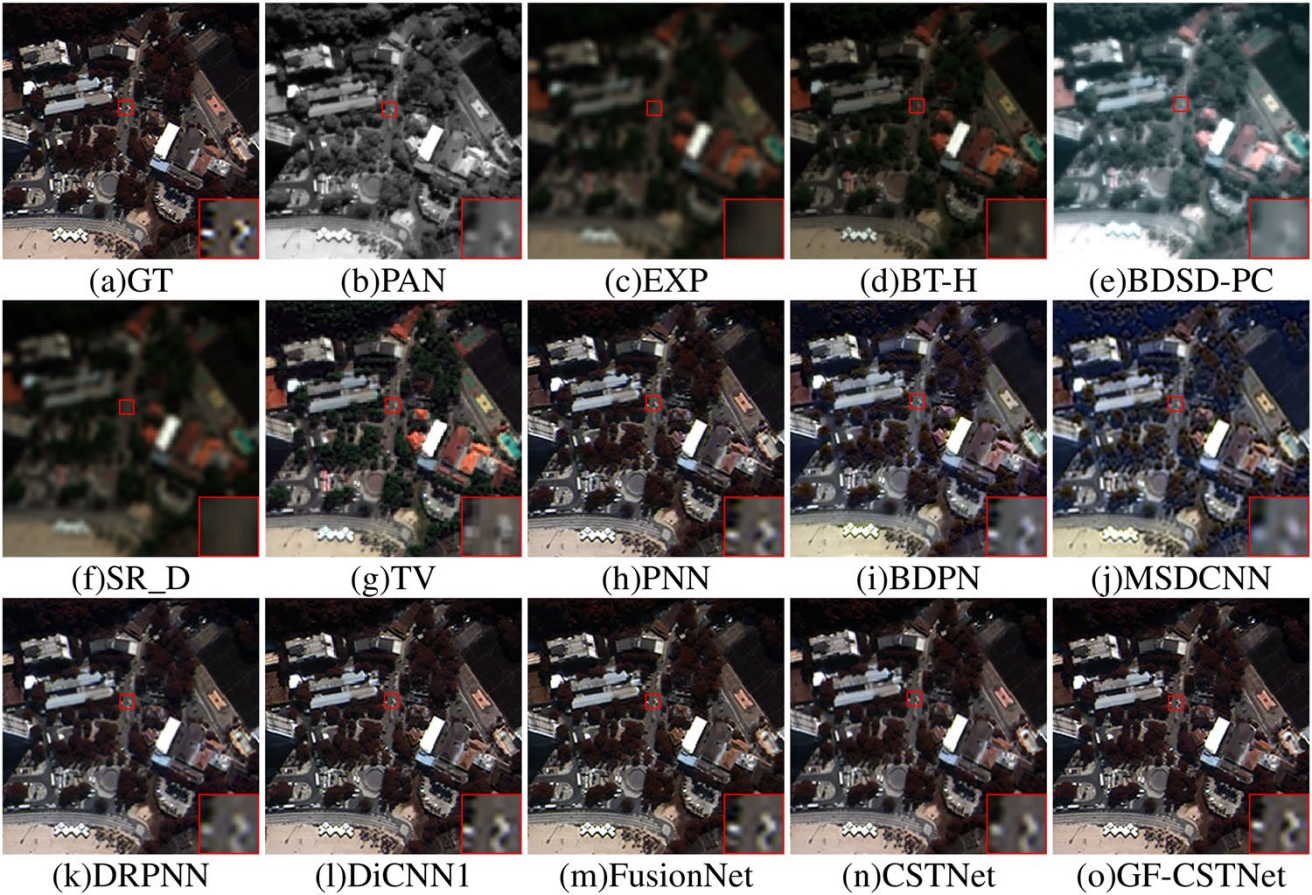
Sharpening results of test Fig. 3 on the WV3 dataset.

**Figure 11:**
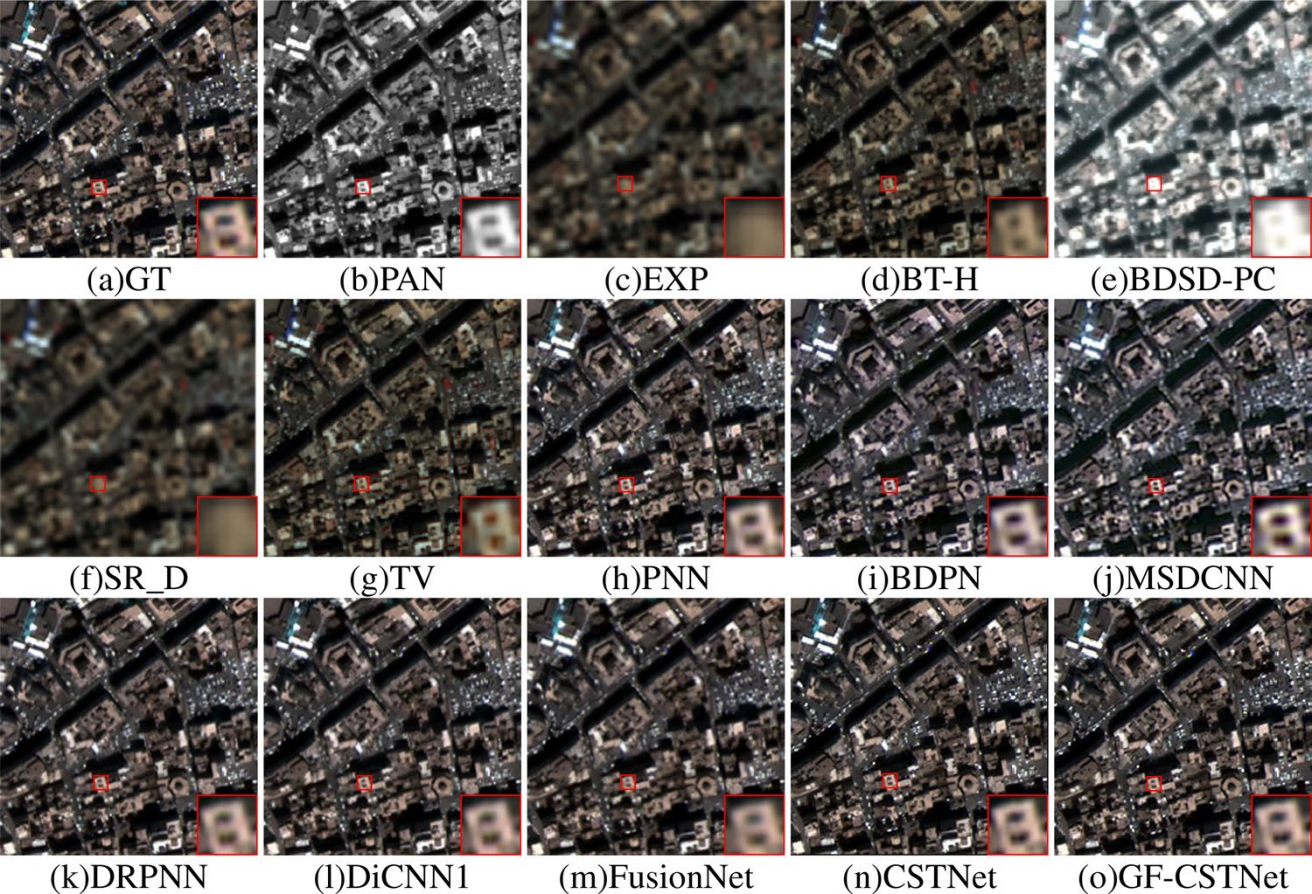
Sharpening results of test Fig. 4 on the WV3 dataset.

The fusion outcomes of various algorithms using the WV3 dataset are illustrated in Figs. 10 and 11. From a subjective perspective, the BT-H (Fig. d) and TV (Fig. g) algorithms exhibit some spectral distortion, and the fusion images lack clarity. The BDSD-PC algorithm (Fig. e) exhibits excessive sharpening and is prone to significant spectral distortion. Although the SR_D algorithm (Fig. f) can effectively preserve spectral features, it can also cause significant spatial distortion, leading to overall ambiguity. The BDPN algorithm (Fig. i) and MSDCNN algorithm (Fig. j) suffer from information loss in either the spectral or spatial domains. The MSDCNN algorithm, however, exhibits more severe overall distortion, leading to a fusion output characterized by evident granularity that differs significantly from the GT image. The fusion results from the PNN algorithm (Fig. h), DRPNN algorithm (Fig. k), DiCNN1 algorithm (Fig. l), FusionNet algorithm (Fig. m), and CSTNet algorithm (Fig. n) more effectively retain the spectral features of the images. However, the boundary texture features are blurred and do not exhibit the same level of clarity as the GT image. In terms of both space and spectrum, the GF-CSTNet (Fig. o) approach presented in this research aligns most closely with the GT image, whether viewed as a whole or in locally magnified areas. Moreover, the edge information is clearer compared to other comparison methods, which achieves better fusion results.

**Table 2:** Evaluation indicators for sharpening results in Test Fig. 2.

Method	Evaluation indicators							
	Q $\uparrow$	ERGAS $\downarrow$	Q4 $\uparrow$	RMSE $\downarrow$	SID $\downarrow$	SAM $\downarrow$	RASE $\downarrow$	SCC $\uparrow$
BT-H	0.9165	1.7580	0.9122	18.5418	0.0057	1.7838	6.2230	0.9102
BDSD-PC	0.9232	1.8871	0.9094	19.7210	0.0056	1.6747	6.6187	0.9078
SR_D	0.9282	1.7963	0.9162	18.8147	0.0135	1.6287	6.3145	0.9093
TV	0.8985	2.2281	0.8537	23.5811	0.0077	2.2892	7.9142	0.9055
PNN	0.9418	1.5061	0.9327	16.0959	0.0037	1.3394	5.4021	0.9550
BDPN	0.9144	1.8055	0.9039	19.1468	0.0067	1.8000	6.4260	0.9374
MSDCNN	0.9271	1.6667	0.9146	17.9355	0.0057	1.5784	6.0195	0.9392
DRPNN	0.9724	1.0306	0.9665	10.9446	0.0025	1.0844	3.6732	0.9745
DiCNN1	0.9519	1.3619	0.9439	14.5386	0.0027	1.2368	4.8794	0.9638
FusionNet	0.9448	1.4621	0.9362	15.5315	0.0028	1.2299	5.2127	0.9602
CSTNet	0.9811	0.8651	0.9765	9.3140	0.0015	0.9401	3.1259	0.9804
GF-CSTNet	**0.9815**	**0.8405**	**0.9766**	**9.0338**	**0.0014**	**0.9338**	**3.0319**	**0.9819**

Significant values are in bold.

The objective assessment metrics for the WV3 dataset are displayed in Tables 3 and 4. Bold numbers indicate the optimal values. As the table clearly shows, the high SAM values of the BT-H, SR_D, and TV algorithms indicate that the fusion image has poor spectral preservation performance. In the BDSD-PC algorithm, the metrics ERGAS, RMSE, RASE, SID, and SAM have the maximum values, while Q and Q8 have the minimum values. This indicates that the fusion results exhibit significant spectral distortion, lack a satisfactory sharpening effect, and bear the least resemblance to the GT image in terms of structural similarity. In the PNN algorithm, the larger SCC number signifies improved spatial performance in the sharpened results. For the BDPN and MSDCNN algorithms show that higher ERGAS values indicate greater overall distortion in the fusion images. The DRPNN, DiCNN1, and FusionNet methods have shown superior performance compared to previously described methods, as indicated by various metrics. This indicates that the sharpened images exhibit notable improvements in both spatial and spectral qualities. The GF-CSTNet method achieves the best results among the eight indexes, followed by the CSTNet method in second place.

**Table 3:** Evaluation indicators for sharpening results in Test Fig. 3.

Method	Evaluation indicators							
	Q $\uparrow$	ERGAS $\downarrow$	Q4 $\uparrow$	RMSE $\downarrow$	SID $\downarrow$	SAM $\downarrow$	RASE $\downarrow$	SCC $\uparrow$
BT-H	0.8773	4.9081	0.8530	65.6844	0.0416	6.3666	15.2849	0.8167
BDSD-PC	0.6471	26.1190	0.3806	345.1671	0.1418	13.3837	101.9327	0.7194
SR_D	0.7456	7.0228	0.7185	93.6517	0.0552	7.6320	21.5246	0.5536
TV	0.8762	5.0553	0.8327	68.9120	0.0444	7.0426	15.6257	0.7802
PNN	0.9582	2.8243	0.9412	38.1448	0.0194	4.6863	8.5813	0.9474
BDPN	0.9103	4.3138	0.8818	57.4421	0.0322	5.9899	14.6656	0.8606
MSDCNN	0.8746	4.9848	0.8243	67.5287	0.0459	7.2541	15.6536	0.7952
DRPNN	0.9621	2.6471	0.9423	35.7721	0.0181	4.5005	8.2106	0.9542
DiCNN1	0.9614	2.6997	0.9455	36.4425	0.0176	4.4873	8.2174	0.9542
FusionNet	0.9679	2.4601	0.9522	33.4912	0.0156	4.1976	7.6316	0.9617
CSTNet	0.9687	2.4042	0.9529	32.7642	0.0151	4.0695	7.4696	0.9642
GF-CSTNet	**0.9715**	**2.2783**	**0.9549**	**30.9701**	**0.0134**	**3.8435**	**7.2316**	**0.9697**

Significant values are in bold.

### Residual experiment

**Figure 12:**
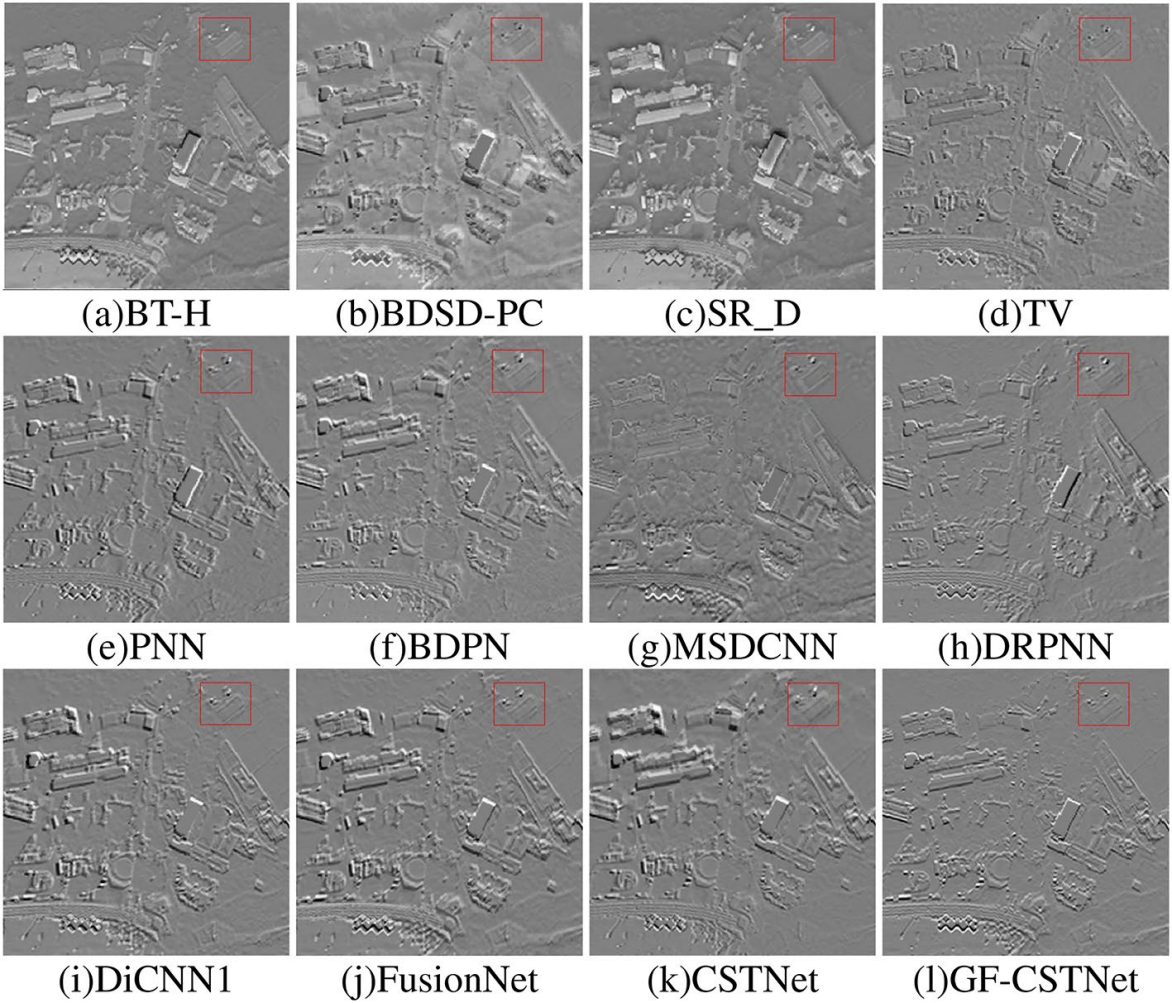
Test Fig. 3 residual chart.

Test Fig. 3 was used as an example in the residual experiment to demonstrate the effective functioning of the suggested method. The average value was determined by computing the difference between the final picture and the one used as a reference. If the resulting image contains less detailed information, it indicates that the algorithm has a higher fusion quality. From Fig. 12, it can be observed that traditional algorithms such as BT-H (Fig. a), BDSD-PC (Fig. b), SR_D (Fig. c), and TV (Fig. d) contain the most detailed information. It indicates that the fusion results are suboptimal, and there is a loss of information within the spectrum and space domains. The residual images from the PNN algorithm (Fig. e), BDPN algorithm (Fig. f), MSDCNN algorithm (Fig. g), DRPNN algorithm (Fig. h), DiCNN1 algorithm (Fig. i), and FusionNet algorithm (Fig. j) show better results than the conventional method. However, there are still distinct texture features in the residual images, indicating that the sharpening results still need improvement. The residual image from the unfiltered CSTNet method (Fig. k) still contains slight texture details, while the filtered GF-CSTNet (Fig. l) method shows the least amount of detail in the residual diagram. This suggests that adopting the approach of enhancing the image through guided filtering first and then performing fusion allows the image to capture more information, resulting in an optimal fusion outcome.

**Table 4:** Evaluation indicators for sharpening results in Test Fig. 4.

Method	Evaluation indicators							
	Q $\uparrow$	ERGAS $\downarrow$	Q4 $\uparrow$	RMSE $\downarrow$	SID $\downarrow$	SAM $\downarrow$	RASE $\downarrow$	SCC $\uparrow$
BT-H	0.9502	3.5292	0.9457	66.8844	0.0148	4.3314	11.5693	0.9452
BDSD-PC	0.6951	25.3615	0.5200	490.1704	0.1274	10.3462	78.4711	0.8983
SR_D	0.7678	7.4053	0.7569	144.6936	0.0221	5.8968	20.9947	0.6228
TV	0.9402	3.9818	0.9358	76.6042	0.0148	5.2824	12.3127	0.9268
PNN	0.9765	2.5353	0.9733	48.1795	0.0087	4.2201	7.6096	0.9620
BDPN	0.9622	3.1284	0.9387	58.0702	0.0126	4.9402	10.6048	0.9522
MSDCNN	0.9435	3.8345	0.9611	71.4832	0.0145	5.4334	12.6215	0.9266
DRPNN	0.9780	2.4555	0.9751	46.8328	0.0094	4.1723	7.1647	0.9640
DiCNN1	0.9788	2.4072	0.9761	45.9886	0.0087	4.0593	7.1062	0.9667
FusionNet	0.9828	2.1069	0.9807	39.3648	0.0064	3.5205	6.8336	0.9796
CSTNet	0.9834	2.0582	0.9815	38.5458	0.0062	3.4577	6.6203	0.9810
GF-CSTNet	**0.9840**	**2.0240**	**0.9820**	**37.9203**	**0.0060**	**3.4165**	**6.5337**	**0.9820**

Significant values are in bold.

### Ablation experiment

The ablation experiments in this section were conducted using the GF2 dataset to validate the feasibility of the suggested approach. The specific details are as follows:

- Ablation regularization (A). On the basis of the FusionNet model (Base), an additional regularization term is introduced to the loss function to mitigate overfitting issues during the model training process.
- Ablation residual learning (B). Based on (1), the modified skeleton network and the residual learning module are considered as a whole for ablation to confirm the effectiveness of residual learning.
- Ablation of attention (C). Building upon (1), the attention module is integrated with the modified skeleton network for ablation to confirm the efficiency of the attention mechanism.
- Ablation of multiple kernels (D). On the basis of (1), open attention is ablated as a way to demonstrate the effectiveness of multi-scale convolution.

**Figure 13:**
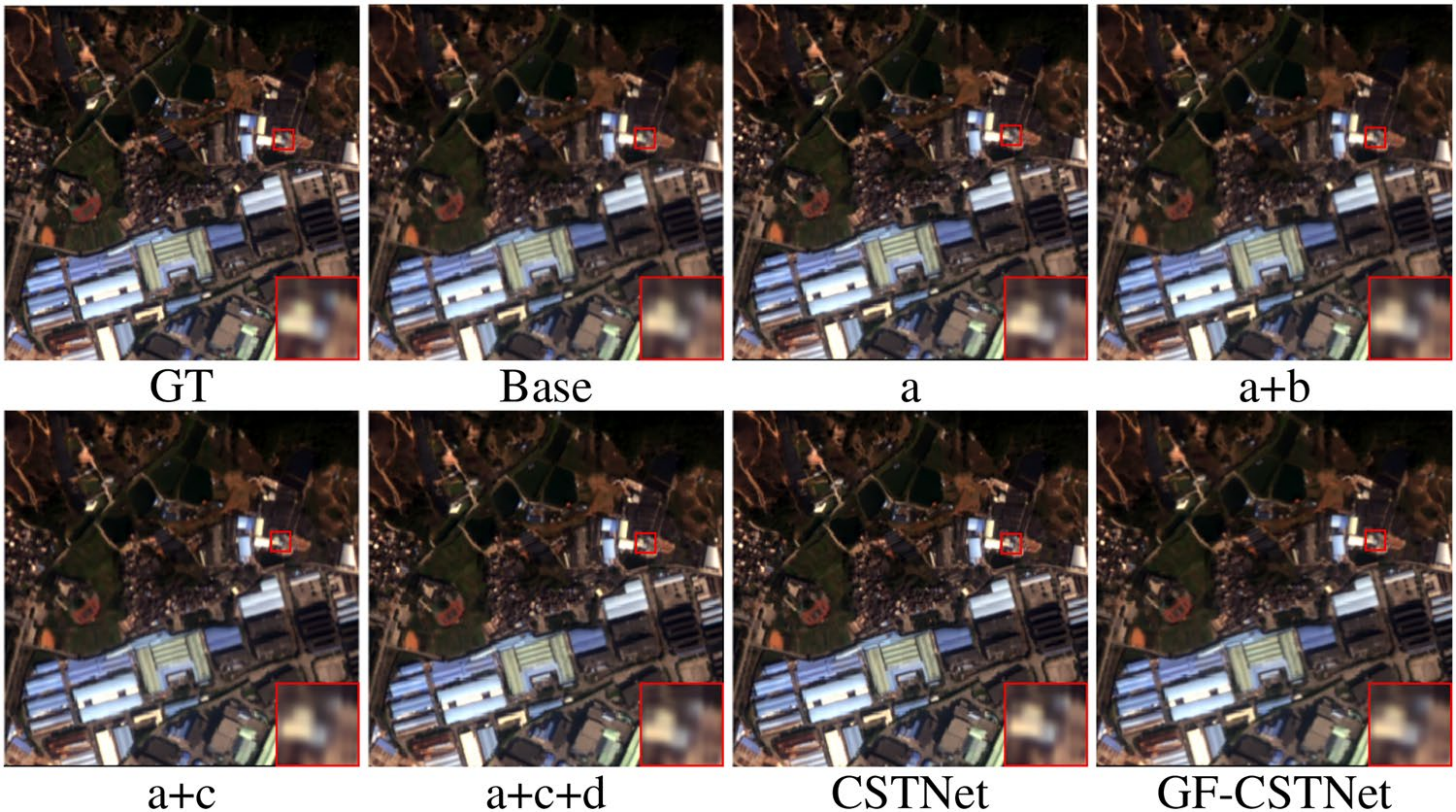
Sharpening results of ablation experiments on the GF2 dataset.

Figure 13 displays the results of the ablation experiment. From a visual standpoint, the sharpening results of a, a + b, a + c, a + c + d, CSTNet, and GF-CSTNet are all superior to Base. Additionally, the sharpening effect of each new module surpasses that of the previous module. Particularly, the GF-CSTNet method, after filtering, is closer to the GT image in terms of spectrum fidelity and spatial information, achieving the most effective sharpening result. Table 5 displays the evaluation metrics. It can be observed that there is an improvement in each of the additional modules compared to the Base. Among them, the GF-CSTNet method, which performs guided filtering operations, achieves optimal values for evaluation metrics, thereby demonstrating the feasibility of the suggested approach.

**Table 5:** Evaluation indicators for ablation experiments on the GF2 dataset.

Method	a	b	c	d	SAM $\downarrow$	ERGAS $\downarrow$	Q4 $\uparrow$	RMSE $\downarrow$	SID $\downarrow$	Q $\uparrow$	RASE $\downarrow$	SCC $\uparrow$
Base					1.2195	1.2672	0.9566	14.4725	0.0026	0.9641	4.5106	0.9692
a	$\checkmark$				1.2110	1.2183	0.9588	14.0123	0.0026	0.9665	4.3671	0.9716
a+b	$\checkmark$	$\checkmark$			1.2078	1.1122	0.9657	12.6006	0.0021	0.9726	3.9271	0.9741
a+c	$\checkmark$		$\checkmark$		1.1228	1.0482	0.9682	11.8019	0.0019	0.9751	3.6782	0.9744
a+c+d	$\checkmark$		$\checkmark$	$\checkmark$	1.0523	0.9874	0.9711	11.2253	0.0017	0.9772	3.4985	0.9798
CSTNet	$\checkmark$	$\checkmark$	$\checkmark$	$\checkmark$	1.0111	0.8820	0.9779	9.9598	0.0015	0.9818	3.1041	0.9819
GF-CSTNet	$\checkmark$	$\checkmark$	$\checkmark$	$\checkmark$	**0.9972**	**0.8385**	**0.9783**	**9.5334**	**0.0014**	**0.9827**	**2.9712**	**0.9841**

Significant values are in bold.

### More discussion

This part conducts more experimental discussion of the suggested approach, focusing mostly on the computational complexity and running time analysis.

**Figure 14:**
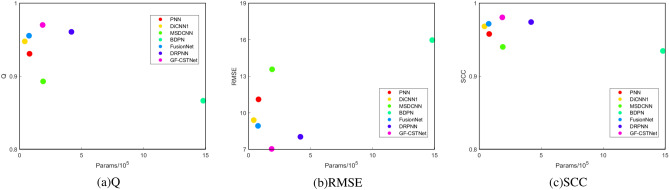
Relationship between the number of parameters and Q, RMSE and SCC under the GF2 dataset.

- Run time Analysis Running efficiency is also a crucial metric when assessing fusion algorithms.This section provides an in-depth analysis of the algorithm’s effectiveness by calculating the average running time of different methods on the GF2 and WV3 datasets. The Tables 6 and 7 show that the method suggested has the least running time and an obvious advantage. Based on comprehensive subjective and objective evaluations, as well as time performance analysis, it is possible to infer that the approach provided in this study performs superiorly.
- Calculation Complexity Analysis For feature maps with input length *n*, in the GF-CSTNet method, the complexity of each layer is mainly affected by the self-attention and convolutional operations due to the use of convolutional modulation blocks to simplify the attention. The overall representation is ${\mathcal {O}}(n^2d+nkd^2)$, where *d* represents the dimension and *k* is the size of the convolution kernel. To further analyze the complexity of the proposed method, Floating Point Operations (Flops) and Params were compared with other methods on the WV3 and GF2 datasets. Where Flops can be used to evaluate the computational effort of the model, which is approximately equivalent to the time complexity, and Params resembles the space complexity of the model. (To facilitate comparison, the conversion follows $1 GFlops=10^9Flops$). As shown in Tables 8 and 9, it can be seen that BDPN has the most parameters and DiCNN1 has the least GFlops. The proposed method uses a dual branch network architecture, which slightly increases the computational cost, but this design also improves the fusion accuracy. To demonstrate the superiority of the proposed method more intuitively, taking the GF2 dataset as an example, Q, RMSE and SCC three indicators were selected to compare the correlation between parameter quantities and indicators. It is evident from Fig. 14 that the sharpening effect of the approach suggested in this research is substantial.

**Table 6:** Average test time of different methods on GF2 dataset.

	PNN	BDPN	DRPNN	DiCNN1	MSDCNN	FusionNet	GF-CSTNet
Average test time/s	0.394	0.390	0.397	0.401	0.406	0.404	**0.388**

Significant values are in bold.

**Table 7:** Average test time of different methods on WV3 dataset.

	PNN	BDPN	DRPNN	DiCNN1	MSDCNN	FusionNet	GF-CSTNet
Average test time/s	0.407	0.401	0.423	0.412	0.430	0.418	**0.397**

Significant values are in bold.

**Table 8:** Comparison of the number of parameters and GFlops on the GF2 dataset.

	PNN	BDPN	DRPNN	DiCNN1	MSDCNN	FusionNet	GF-CSTNet
Parameters	80420	1481273	418365	42180	189852	76324	186213
GFlops	0.33	0.38	1.71	0.17	0.78	0.31	1.71

**Table 9:** Comparison of the number of parameters and GFlops on the WV3 dataset.

	PNN	BDPN	DRPNN	DiCNN1	MSDCNN	FusionNet	GF-CSTNet
Parameters	104360	1487069	433465	46792	228556	78632	203209
GFlops	0.43	0.38	1.77	0.19	0.93	0.32	1.82

## Conclusion

This paper introduces a novel pansharpening method named GF-CSTNet, which combines CSPNet with Transformer, for enhancing images from remote sensors. In this method, guided filtering is applied to enhance the multispectral image initially. Subsequently, the Transformer structure is integrated into CSPNet, and a new multi-scale convolutional modulator block, along with a parameter-heavy block, is designed by drawing inspiration from the Conv2Former method and the RepVGG method. This design enables the model to extract more information from diverse receptive fields, enhancing the spatial and spectral resolution of the fusion image. Moreover, the GF-CSTNet approach optimizes the direct difference method. It introduces a residual learning block incorporating attention, further improve the overall quality of the fusion image. Compared to alternative approaches, the proposed GF-CSTNet method significantly enhances fusion excellence, as evidenced by its validation on the GF2 and WV3 datasets. It achieves optimal results across multiple objective evaluation metrics. Additionally, residual experiments and ablation experiments further affirm the efficacy of the GF-CSTNet approach.

## Data Availability

The datasets used during the current study are available from the corresponding author on reasonable request. The code is available on GitHub (https://github.com/Liu-9911/GF-CSPNet).
